# Structural basis of SARS-CoV-2 polymerase inhibition by nonnucleoside inhibitor HeE1-2Tyr

**DOI:** 10.1073/pnas.2419854122

**Published:** 2025-03-04

**Authors:** Florian Kabinger, Valerie Doze, Jana Schmitzová, Michael Lidschreiber, Christian Dienemann, Patrick Cramer

**Affiliations:** ^a^Department of Molecular Biology, Max Planck Institute for Multidisciplinary Sciences, Göttingen 37077, Germany

**Keywords:** SARS-CoV-2, RNA-dependent RNA polymerase, cryo-EM, non-nucleoside inhibitor

## Abstract

Targeting the RNA-dependent RNA polymerase (RdRp) of SARS-CoV-2 with small molecules is a promising therapeutic strategy against COVID-19, but potent and safe inhibitors are lacking. HeE1-2Tyr, a nonnucleoside inhibitor of Dengue virus RdRp, was also shown to inhibit SARS-CoV-2 RdRp in vitro and to have antiviral activity in cells, but the underlying mechanism remains unclear. Here, we elucidate the molecular mechanism of HeE1-2Tyr-mediated SARS-CoV-2 RdRp inhibition. Biochemical assays confirm that HeE1-2Tyr inhibits RdRp with an IC_50_ of 5 µM and show that it competes with RNA binding to RdRp in vitro. Structural analysis using cryo-EM reveals that a stack of three HeE1-2Tyr molecules binds to the RNA binding site of RdRp. The identification of the conserved HeE1-2Tyr binding site and its intriguing inhibition mechanism of three stacked molecules that outcompete RNA may facilitate further development of pan-corona nonnucleoside inhibitors.

## Significance

Despite significant progress in overcoming the COVID-19 pandemic, SARS-CoV-2 continues to spread and new variants are emerging, highlighting the need for effective antivirals. The viral replication machinery, the RNA-dependent RNA polymerase (RdRp), is a promising therapeutic target, but potent inhibitors are lacking and their development is often hampered by a lack of mechanistic understanding. Here, we present biochemical data and an atomic structure of SARS-CoV-2 RdRp bound to a stack of three HeE1-2Tyr molecules, revealing that HeE1-2Tyr inhibits SARS-CoV-2 RdRp by competing with RNA binding. Together with the high conservation of the HeE1-2Tyr binding site across coronaviruses, these results provide a basis for the potential development of better RdRp inhibitors not only against SARS-CoV-2, but also against coronaviruses in general.

Severe acute respiratory syndrome coronavirus 2 (SARS-CoV-2) is the causative agent of COVID-19, a disease that has claimed more than 7 million lives worldwide since its outbreak in 2020 (1, 2). SARS-CoV-2 is a positive-sense single-stranded RNA virus that encodes 16 nonstructural proteins (nsp) that facilitate the viral life cycle (3). The RNA-dependent RNA polymerase (RdRp) consists of the polymerase subunit nsp12 and the accessory subunits nsp7 and nsp8 (4, 5). The RdRp is essential for replication and transcription of the viral genome and is therefore a highly promising drug target. Inhibition of RdRp with small molecules prevents the production of viral RNA and stops the propagation of the virus (6–8).

RdRp inhibitors can be broadly classified into nucleoside and nonnucleoside inhibitors. Nucleoside inhibitors, also referred to as nucleoside analogues, mimic the naturally occurring substrate of polymerases, nucleoside triphosphates (NTPs), or their precursors (9). In previous studies, we and others have characterized the mode of action of the nucleoside inhibitors Remdesivir (10, 11) and Molnupiravir (12, 13), which received an Emergency Use Authorization (EUA) for clinical usage against SARS-CoV-2 (14, 15). However, due to the chemical nature of nucleoside inhibitors, they have numerous disadvantages related to their pharmacodynamics and -kinetics. For example, Remdesivir and Molnupiravir are disputed due to issues with efficacy and specificity, respectively (16–18). Nonnucleoside inhibitors are structurally distinct from nucleosides or NTPs and usually have a binding site outside the NTP binding pocket of the polymerase. Thus, such molecules are not constrained by their similarity to nucleosides and could help to address the strong need for potent and safe RdRp inhibitors. Unfortunately, despite global efforts to find suitable small molecules since the beginning of the pandemic, promising nonnucleoside inhibitors are still lacking (19).

Many in silico studies have led to the nomination of numerous potential RdRp inhibitors (20–26). However, very few of these molecules have shown reproducible inhibition in vitro. In addition, the further development of discovered small molecule inhibitors is often hampered by a lack of mechanistic insight and structural data of RdRp–inhibitor complexes (19). There is only one published structure of RdRp bound to a nonnucleoside inhibitor called suramin (27). Suramin binds near the RNA binding site of RdRp and displaces RNA. However, Suramin is a very large repurposed molecule that contains six sulfonic acid groups and is known for its promiscuity (28–30). Therefore, it is not possible to use Suramin to treat COVID-19.

**Fig. 1.**
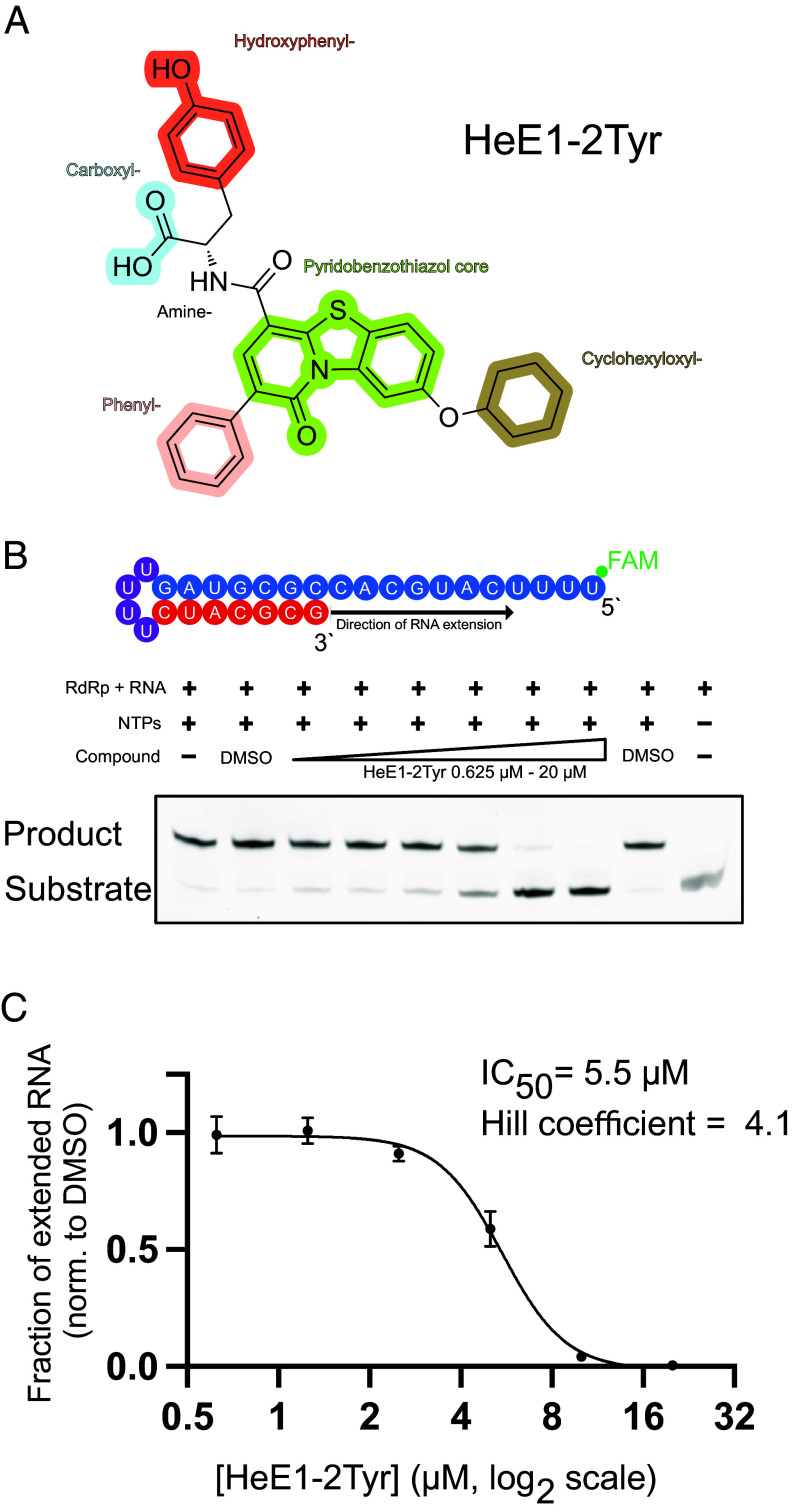
HeE1-2Tyr is a potent nonnucleoside inhibitor of SARS-CoV-2 RdRp. (*A*) Chemical structure of HeE1-2Tyr; relevant chemical functional groups are highlighted and labeled. (*B*) *Top*: minimal RNA substrate that folds into a hairpin with “template” (blue), “product” (red), and “loop” (purple) regions. The RNA is fluorescently labeled with 5′ 6-carboxyfluorescein (FAM). *Bottom*: HeE1-2Tyr inhibits RNA extension by RdRp in a dose-dependent manner. (*C*) Quantification of the fraction of extended RNA of HeE1-2Tyr containing reactions normalized to the DMSO control. Mean ± SD of independent triplicate measurements are shown. RdRp concentration 125 nM, HeE1-2Tyr concentration is represented on a logarithmic scale with base 2. Half maximal inhibitory concentration (IC_50_) and Hill coefficient were determined by fitting a nonlinear dose–response curve. A Hill coefficient greater than 1 means that the binding of one molecule facilitates the binding of other molecules, indicating positive cooperativity of HeE1-2Tyr binding to RdRp.

HeE1-2Tyr is a pyridobenzothiazole derivative that was first described as a flavivirus RdRp inhibitor (chemical structure shown in Fig. 1*A*) (31). HeE1-2Tyr inhibits Dengue virus polymerase (NS5 protein) in a dose-dependent manner in vitro and shows antiviral effects in cell assays (31). The mode of action remains disputed, but it seems likely that HeE1-2Tyr binds directly to NS5 and prevents its activity (31–33). Later, it was reported that HeE1-2Tyr is also effective against the RdRp of SARS-CoV-2 in vitro and in cell culture experiments (34). However, the binding site of HeE1-2Tyr and mechanistic insights into HeE1-2Tyr-mediated RdRp inhibition remain unknown.

Here, we elucidate the mode of action underlying HeE1-2Tyr-mediated inhibition of SARS-CoV-2 RdRp. Using RNA elongation and fluorescence polarization assays, we show that HeE1-2Tyr is a competitive inhibitor of RNA binding to SARS-CoV-2 RdRp and thereby prevents RNA extension. Building on this, we determine the high-resolution cryo-EM structure of HeE1-2Tyr bound to RdRp, which reveals that three molecules of HeE1-2Tyr are stacked on each other and are bound to the highly conserved RNA binding site. Our systematic biochemical and structural results not only provide detailed insights into the mode of action of HeE1-2Tyr, but also suggest HeE1-2Tyr as a starting molecule for the development of future pan-corona nonnucleoside RdRp inhibitors.

## Results

### HeE1-2Tyr Is a Potent Nonnucleoside Inhibitor of SARS-CoV-2 RdRp.

To recapitulate the reported inhibitory effect of HeE1-2Tyr (Fig. 1*A*) against SARS-CoV-2 RdRp (34) in our biochemical system, we used RNA elongation assays as described (5) with recombinantly produced RdRp and a minimal RNA hairpin substrate (Fig. 1*B*, *Methods*). RdRp was incubated with RNA and up to 20 µM HeE1-2Tyr, and the reaction was started by the addition of NTPs. RNA extension by RdRp was then analyzed by detecting the 5′ fluorescent label of the RNA hairpin after allowing RNA extension for 5 min.

Without HeE1-2Tyr, the RNA substrate was readily extended within 5 min (Fig. 1*B*). At HeE1-2Tyr concentrations of 0.625 to 2.5 µM, most of the RNA substrate was extended. However, when adding 5 µM or more HeE1-2Tyr, we observed a dose-dependent inhibitory effect that almost completely abolished RdRp activity at concentrations above 10 µM. Quantitative analysis of three independent experiments and the fit of a dose–response curve resulted in an IC_50_ of 5.5 µM (Fig. 1*C*), which is five times lower than reported before (34). This is likely due to the higher activity of the coexpressed RdRp used here, which allowed us to use significantly lower protein concentration in the biochemical assay compared to RdRp reconstituted from individually expressed subunits (*SI Appendix*, Fig. S1). Additionally, we determined a Hill coefficient of 4.1, suggesting positive cooperativity of RdRp inhibition by HeE1-2Tyr.

In summary, our results demonstrate that HeE1-2Tyr is a potent nonnucleoside inhibitor against RdRp of SARS-CoV-2 in vitro, consistent with prior reports (34). Combining the minimal RNA substrate with highly active coexpressed RdRp provides a versatile system that allows us to further study the molecular mechanisms of SARS-CoV-2 RdRp inhibition by HeE1-2Tyr.

### HeE1-2Tyr Interferes with RNA Binding to SARS-CoV-2 RdRp.

**Fig. 2.**
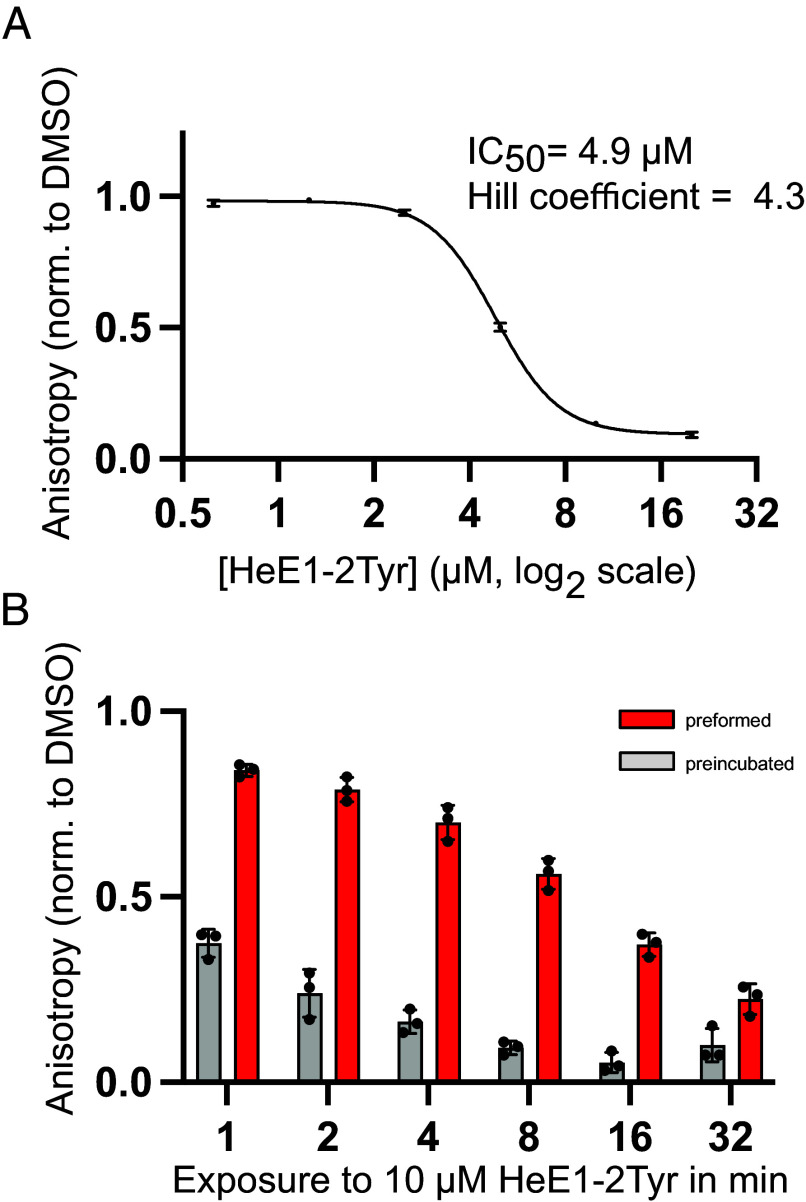
HeE1-2Tyr interferes with RNA binding to SARS-CoV-2 RdRp. (*A*) HeE1-2Tyr inhibits the interaction between RdRp and RNA in a dose-dependent manner. Quantification of the decrease in fluorescence anisotropy of RdRp samples containing HeE1-2Tyr normalized by the DMSO control. Mean ± SD of independent triplicate measurements are shown. Some error bars are too small to be depicted, raw values are provided in the source data. RdRp concentration 125 nM, HeE1-2Tyr concentration is represented on a logarithmic scale with base 2. Half maximal inhibitory concentration (IC_50_) and Hill coefficient were determined by fitting a nonlinear dose–response curve. (*B*) 10 µM HeE1-2Tyr have significantly stronger inhibitory effect when added before the RNA. RdRp (125 nM) was either preformed with RNA and then exposed to HeE1-2Tyr (red) or preincubated with HeE1-2Tyr and then allowed to interact with RNA (gray). Total time of exposure depicted on a logarithmic scale with base 2; individual data points and boxes represent mean ± SD of independent triplicate measurements.

Next, we investigated how HeE1-2Tyr inhibits SARS-CoV-2 RdRp. To test whether HeE1-2Tyr has an effect on the RNA binding properties of RdRp we established a fluorescence polarization (FP) assay utilizing the same fluorescently labeled RNA hairpin scaffold as in the transcription assay (*SI Appendix*, Fig. S2*A*). Since the polarization of the emitted fluorescence is proportional to the hydrodynamic radius of the fluorescently tagged molecule (35), the change of FP was used to calculate the fraction of RNA bound to RdRp (*Methods*). The FP assay showed that coexpressed RdRp binds the RNA scaffold with a K_D_ of 75 nM (*SI Appendix*, Fig. S2*B*). Addition of HeE1-2Tyr caused a dose-dependent reduction in RNA binding to RdRp, where RNA binding was nearly abolished at HeE1-2Tyr concentrations above 10 µM (Fig. 2*A*). Accordingly, quantitative analysis of three independent experiments revealed that HeE1-2Tyr inhibits the interaction of RdRp and RNA with an IC_50_ of 4.9 µM and a Hill coefficient of 4.3. These numbers are almost identical to the results obtained from the RNA extension assay (Fig. 1*C*), suggesting that HeE1-2Tyr inhibits RNA extension by interfering with RNA binding to RdRp.

To study the interference between RNA and HeE1-2Tyr in more detail, we repeated the FP assay while varying the order of addition of RNA and HeE1-2Tyr. For preincubated samples, RdRp and HeE1-2Tyr were incubated together before RNA was added. Whereas for preformed samples, RdRp was allowed to form a complex with RNA before HeE1-2Tyr was added. Comparison of the fraction of RdRp-bound RNA in the FP assay between preincubated and preformed reactions showed that 10 µM HeE1-2Tyr is significantly more effective when added before the RNA (Fig. 2*B*). The difference is most pronounced for short incubation times and diminishes over time. Based on these results, we further tested whether the rate of disruption of a preformed RdRp–RNA complex is dependent on the concentration of HeE1-2Tyr. Indeed, the time needed to disrupt 50% of the RdRp–RNA complex was significantly shorter at 20 µM than at 5 µM HeE1-2Tyr (*SI Appendix*, Fig. S3).

Altogether, we show that HeE1-2Tyr at 5 µM or higher can displace RNA from RdRp irrespective of the order of addition.

### Cryo-EM Structural Analysis of HeE1-2Tyr Binding to RdRp.

Next, we set out to determine the exact binding mode of HeE1-2Tyr to RdRp using single particle cryo-EM. Based on our finding that HeE1-2Tyr interferes with RNA binding to RdRp, we needed to prepare a stable RdRp complex in the absence of RNA. This has been difficult with RdRp reconstituted from individually expressed subunits (36) because the yield of fully assembled RdRp complex is low (*SI Appendix*, Fig. S4). The coexpressed and copurified RdRp (37) that we used for our biochemical experiments, however, shows a significantly higher proportion of fully assembled RdRp (*SI Appendix*, Fig. S4), which makes it highly suitable for structural investigation of RdRp–inhibitor complexes without RNA.

We acquired cryo-EM datasets of RdRp without RNA, but in presence of 50 µM HeE1-2Tyr, as well as RdRp with 0.5% DMSO as a solvent control. For the HeE1-2Tyr containing dataset, we first identified 1.65 million good particles by two rounds of heterogeneous refinements in cryoSPARC (38) with a published RdRp volume [EMD-30572 (27)] and three volumes created from junk particles (*SI Appendix*, Fig. S5). The good particles were then subjected to 3D classification without image alignment in Relion (39) to separate fully assembled RdRp from smaller subcomplexes, which resulted in ~300,000 good particles of the RdRp complex.

During 3D classification, we identified a particle species containing extra density in the RNA binding site of RdRp. To clearly separate particles with and without the extra density, we performed focused 3D classification without image alignment. This resulted in ~200,000 RdRp particles containing an extra density, which could be refined to 3.0 Å. Except for the last step, the DMSO control dataset was analyzed employing a similar strategy, yielding a 3.1 Å reconstruction (*SI Appendix*, Fig. S5, *Methods*). The overall conformation of RdRp in both reconstructions is very similar to previously determined structures (27, 40). For the reconstruction of RdRp in presence of HeE1-2Tyr, we docked and refined a published molecular model of RdRp [PDB: 6M71 (40)] comprising nsp12, nsp7, and two copies of nsp8, which yielded high model quality scores (*SI Appendix*, Table S1).

### HeE1-2Tyr Binds to RdRp as a Stack of Three Molecules.

**Fig. 3.**
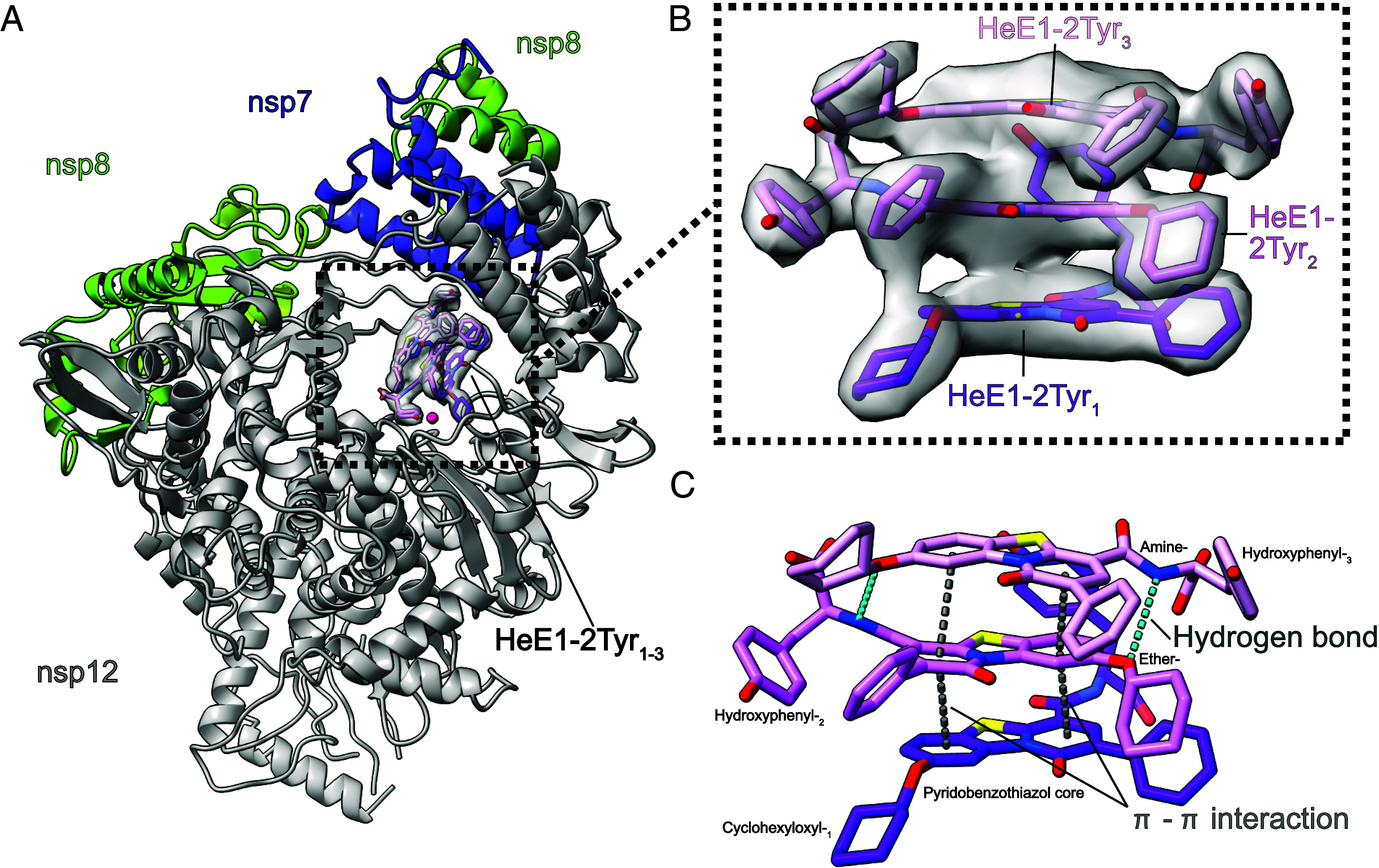
Structure of RdRp bound to a stack of three HeE1-2Tyr molecules. (*A*) Overview of the cryo-EM structure of RdRp bound to a stack of three HeE1-2Tyr molecules (shades of purple). Cryo-EM density of HeE1-2Tyr molecules is shown. The active site Mg^2+^ ion is modeled based on ref. 41. and indicated by a magenta sphere. (*B*) Zoom-in of the density of HeE1-2Tyr molecules with fitted compound models. The density could be unambiguously interpreted by three stacked HeE1-2Tyr molecules, HeE1-2Tyr_1-3_ are shown in violet, pink, and light red, respectively. (*C*) Stacking of the HeE1-2Tyr molecules is driven by intercompound π–π interactions (dark gray) and hydrogen bonds (cyan).

To compare the cryo-EM maps derived from the dataset of the RdRp-DMSO control and RdRp in the presence of HeE1-2Tyr, we calculated a difference map of both (*Methods*). This revealed a defined extra density in the RNA binding site of nsp12 for the reconstruction from the compound-containing sample (*SI Appendix*, Fig. S6*A*). The density in this area is locally resolved to 2.8 Å and shows clear features for bulky chemical groups. The extra density is larger than expected for a single HeE1-2Tyr molecule and shows a three-layered arrangement (*SI Appendix*, Fig. S6*B*). Based on the asymmetric chemical structure of HeE1-2Tyr (Fig. 1*A*) and the high local resolution, we could unambiguously model three stacked molecules of HeE1-2Tyr (Fig. 3*B*), which we refer to as HeE1-2Tyr_1–3_, with HeE1-2Tyr_1_ being the molecule closest to the active site of nsp12.

The stacking between the three HeE1-2Tyr molecules is driven by the aromatic pyridobenzothiazole moieties of the compound molecules, which are arranged on top of each other with a planar antiparallel geometry (Fig. 3*C*). The spacing between the molecule planes is 3.6 Å for HeE1-2Tyr_1/2_ and 3.4 Å for HeE1-2Tyr_2/3_, which is in accordance with π–π interactions. (42, 43) HeE1-2Tyr_1_ and HeE1-2Tyr_3_ are in the same orientation with the phenyl-group pointing toward the RdRp thumb domain (Fig. 3*C*), whereas HeE1-2Tyr_2_ is flipped by 180°. The conformations of the sidechains of the three HeE1-2Tyr molecules differ in order to allow for interactions within the stack and with RdRp (*SI Appendix*, Fig. S7). Besides π–π interactions, HeE1-2Tyr_2_ and HeE1-2Tyr_3_ are stabilized by the formation of two hydrogen bonds (H-bond) between the oxygen of the ether group and the nitrogen of the amine group of each molecule (Fig. 3*C*).

In summary, we solved the cryo-EM structure of SARS-CoV-2 RdRp in complex with HeE1-2Tyr. The compound is bound as a stack of three HeE1-2Tyr molecules that is stabilized by intercompound π–π and H-bond interactions. Three-molecule stacking of HeE1-2Tyr within SARS-CoV-2 RdRp represents an intriguing binding mode of a small-molecule RdRp inhibitor.

### The HeE1-2Tyr Stack Is Stabilized by an Arginine Bracket.

**Fig. 4.**
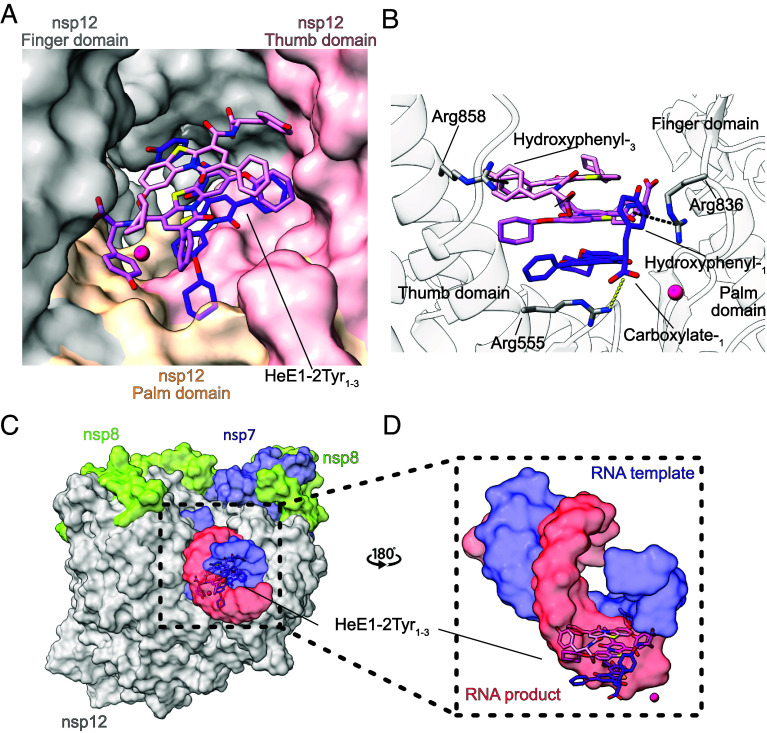
An arginine bracket stabilizes HeE1-2Tyr inside the RNA binding site of RdRp. (*A*) HeE1-2Tyr molecules shown inside the RNA binding site of RdRp. Finger, palm, and thumb domain of nsp12 are shown in gray, yellow, and red surface representation, respectively. Molecules of HeE1-2Tyr_1–3_ are depicted in violet, pink, and light red, respectively. The active site Mg^2+^ ion is indicated by a magenta sphere. (*B*) The arginine bracket stabilizes the stack of HeE1-2Tyr molecules inside the RNA binding site of RdRp, an ionic bond (yellow) is formed between carboxylate of HeE1-2Tyr_1_ and the guanidinium group of Arg555. Two cation–π interactions (black) are observed between the hydroxyphenyl-_1_ and the guanidinium group of Arg837, and hydroxyphenyl-_3_ and the guanidinium group of Arg858. Nsp12 domains and relevant chemical functional groups are labeled. The active site Mg^2+^ ion is indicated by a magenta sphere. (*C*) Structural overlay of RdRp bound to HeE1-2Tyr or RNA [PDB ID: 6yyt (5)]. The RNA template (blue) and product (red) are shown as semitransparent surface representation. HeE1-2Tyr_1–3_ are violet, pink, and light red, as before. (*D*) The stack of three HeE1-2Tyr molecules sterically clashes with RNA, rendering the interaction mutually exclusive. Same color code as (*C*), view is rotated by 180° relative to (*C*).

The stack of three HeE1-2Tyr molecules binds directly into the RNA binding site of nsp12 between the finger, palm, and thumb domain (Fig. 4*A*). The three HeE1-2Tyr molecules are bound to RdRp by several types of noncovalent interactions including an ionic bond, hydrogen bonds, and cation–π interactions (*SI Appendix*, Fig. S8). HeE1-2Tyr_1_ has the largest surface area exposed to RdRp and consequently has the most interactions with nsp12 (Fig. 4*B*). The carboxylate of HeE1-2Tyr_1_ and the guanidinium group of Arg836 are 3.3 Å apart, which suggests the formation of an ionic bond (Fig. 4*B*). In addition, Ser814 is predicted to form a hydrogen bond with the tertiary amine of HeE1-2Tyr_1_ (Fig. 4*B*). Besides that, it is known from other protein-ligand structures that guanidinium groups of arginine residues can form relatively stable cation–π interactions with aromatic systems (44, 45). Such a cation–π interaction is formed between the hydroxyphenyl moiety of HeE1-2Tyr_1_ and the guanidinium group of Arg555 (Fig. 4*B*). HeE1-2Tyr_2_ has, apart from Van-der-Waals interactions and one potential weak polar interaction between its hydroxyphenyl moiety and a nitrogen in the backbone of Gly590, no significant interactions with RdRp. This suggests that HeE1-2Tyr_2_ is mostly stabilized by the π–π interactions with the neighboring HeE1-2Tyr_1_ and HeE1-2Tyr_3_ molecules (Fig. 3*C*). HeE1-2Tyr_3_, which is the molecule furthest away from nsp12, forms a cation–π interaction with Arg858 of RdRp (Fig. 4*B*).

From these interactions, the three arginines 555, 836, and 858 appear to be most important for the interaction between the HeE1-Tyr molecules and RdRp, and we therefore call them the arginine bracket. Interestingly, the arginine bracket residues also interact with RNA in the structure of replicating RdRp (5). This suggests that RNA and HeE1-2Tyr binding to RdRp are mutually exclusive for steric reasons. Indeed, when the RdRp–RNA and RdRp-HeE1-2Tyr structures are superimposed, a steric clash between the stack of HeE1-2Tyr molecules and the RNA becomes apparent (Fig. 4 *C* and *D*). This readily explains our biochemical data and further confirms that HeE1-2Tyr is a competitive inhibitor of RNA binding to SARS-CoV-2 RdRp.

In summary, the main interactions of the He1-2Tyr stack with RdRp are mediated through HeE1-2Tyr_1_ and HeE1-2Tyr_3_, whereas HeE1-2Tyr_2_ is predominantly stabilized by π–π interactions and hydrogen bonds within the stack. Three RdRp arginine residues form an arginine bracket that stabilizes the stack of HeE1-2Tyr molecules within the RdRp RNA binding site by the formation of two cation–π and one ionic interaction. Thus, our atomic structure of RdRp bound by a stack of three HeE1-2Tyr molecules explains how the inhibitor interferes with RNA binding and transcription of SARS-CoV-2 RdRp.

### RNA and HeE1-2Tyr Both Stabilize the RdRp Complex.

**Fig. 5.**
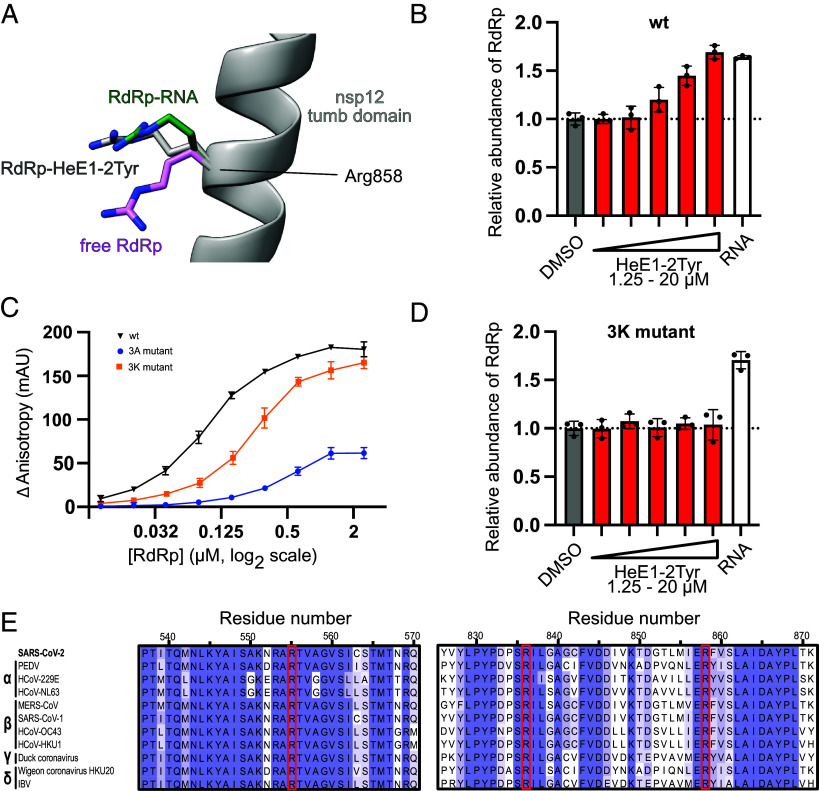
The arginine bracket is important for RNA and HeE1-2Tyr binding to RdRp. (*A*) Comparison of the position of Arg858 in RdRp bound to RNA [green, PDB ID: 6yyt (5)], HeE1-2Tyr (gray) and without RNA [pink, PDB ID: 6M71 (40)]. (*B*) HeE1-2Tyr stabilizes the RdRp complex similar to RNA. Mass photometry-based quantification of the abundance of RdRp (300 nM) in samples containing HeE1-2Tyr (red) or RNA (white) normalized to the DMSO control (gray). Individual data points and boxes represent mean ± SD of independent triplicate measurements. (*C*) Analysis of the RNA binding properties of RdRp wild type (black), the 3 K mutant (nsp12 R555K, R836K, R858K; yellow), and 3A mutant (nsp12 R555A, R836A, R858A; blue) using the fluorescence polarization assays as in Fig. 2*A*. Mean ± SD of independent triplicate measurements are shown. RdRp concentration is represented on a logarithmic scale with base 2. (*D*) 3 K mutant RdRp is stabilized by RNA but not by HeE1-2Tyr. Same experimental setup and color code as in (*C*). (*E*) Sequence conservation of the arginine bracket in different coronaviruses. Multiple sequence alignment (MSA) for the ORF1ab of SARS-CoV-2, Porcine epidemic diarrhea virus (PEDV), Human coronavirus 229E (HCoV-229E), Human coronavirus NL63 (HCoV-NL63), Middle East respiratory syndrome-related coronavirus (MERS-CoV), Severe acute respiratory syndrome coronavirus 1 (SARS-CoV-1), Human coronavirus OC43 (HCoV-OC43), Human coronavirus HKU1 (HCoV-HKU1), Duck corona virus and widgeon corona virus HKU20, and Chicken infectious bronchitis virus (IBV). Alignment colored by sequence conservation, residues of the arginine bracket are highlighted with a red box. The coronavirus family is indicated at the left.

To investigate whether binding of the HeE1-2Tyr molecule stack induces conformational changes in RdRp, we compared our structure with structures of free RdRp [PDB ID: 6m71 (40)] and RdRp bound to RNA [PDB ID: 6yyt (5)]. All three residues of the arginine bracket show a substantial rearrangement compared to the free RdRp structure to accommodate the ligand molecules. Arg858, for example, adopts a conformation more similar to that of RNA-bound RdRp (Fig. 5*A*). Interestingly, the arginine bracket residues are also better resolved in the cryo-EM density of the HeE1-2Tyr-bound RdRp when compared to the DMSO control, suggesting that these residues adopt a more defined conformation when they interact with HeE1-2Tyr.

Binding of HeE1-2Tyr structurally connects the finger, palm, and thumb domains of RdRp using the same arginine residues as involved in RNA binding. Related to that, we and others observed that the RdRp complex is more stable when bound to RNA (36). Thus, we hypothesized that HeE1-2Tyr might also have a stabilizing effect on the RdRp complex. To investigate this, we first incubated the RdRp complex with increasing concentrations of HeE1-2Tyr ranging from 1.25 µM to 20 µM and then measured the fraction of fully assembled RdRp using mass photometry (Fig. 5*B*). Indeed, HeE1-2Tyr had a dose-dependent stabilizing effect on RdRp as evident by the increase of the fraction of fully assembled RdRp in the sample. To compare the effect with that of RNA, we next incubated RdRp with 1.25 µM RNA and then analyzed the fraction of RdRp and free nsp12 in the sample by mass photometry. RNA binding stabilized RdRp to a similar extent as binding of HeE1-2Tyr (Fig. 5*B*). In summary, when RdRp binds to HeE1-2Tyr or RNA, significantly more fully assembled RdRp comprising nsp7, nsp8, and nsp12 was observed. We therefore propose that the arginine bracket is important for stabilizing RdRp in the presence of HeE1-2Tyr or RNA as it interacts with both.

### The Arginine Bracket Is Required for RNA Binding and Is Conserved Across Coronaviruses.

Next, we further characterized the binding site of HeE1-2Tyr and the importance of the arginine bracket for HeE1-2Tyr and RNA binding. For this purpose, we mutated the residues Arg555, Arg836, and Arg858 to lysines (3 K mutant) or alanines (3A mutant). The 3 K mutant should retain the positive charges of the arginine bracket required for RNA binding, but might show defects in the formation of cation–π interactions with HeE1-2Tyr due to the changed side chain geometry (44, 46). In contrast, the 3A mutant should abolish all interactions with RNA and HeE1-2Tyr. Expression, purification, and biophysical behavior of the mutants were virtually identical to the wild type RdRp, confirming that the mutant RdRp complexes were correctly formed. When analyzing the RNA binding properties of the mutant RdRp complexes using the FP assay, we observed that the 3 K mutant is still able to bind RNA, but with twofold decreased affinity compared to wild type, whereas the 3A mutant shows very weak RNA binding (Fig. 5*C*). The reduction in RNA binding and potential catalytic defects are further reflected by a decreased transcriptional activity of the 3 K and 3A mutants compared to wild type (*SI Appendix*, Fig. S9). This is consistent with a previous study showing the involvement of Arg555 in substrate selection and positioning (47). Thus, the arginine bracket residues are important for RNA binding and transcription of SARS-CoV-2 RdRp.

Intrigued by our previous results which suggested a stabilizing effect of HeE1-2Tyr on wild type RdRp, we tested the fraction of fully assembled 3 K mutant RdRp in the presence of HeE1-2Tyr or RNA using mass photometry. In contrast to wild type RdRp, HeE1-2Tyr had no effect on the stability of the 3 K mutant, while RNA was still able to stabilize the complex (Fig. 5*D*). Taken together, this suggests that HeE1-2Tyr is unable to bind to RdRp when the residues of the arginine bracket are converted to lysines, and therefore provides further validation of the compound binding site.

Because of the importance of the arginine bracket for SARS-CoV-2 function, we further investigated the sequence conservation of the arginine residues in different coronaviruses. Our analysis contains representatives from all four coronavirus families, including pandemic-causing viruses such as SARS-CoV-1 or MERS-CoV, seasonal human coronaviruses, and animal coronaviruses (Fig. 5*E*). While the overall sequence identity of the analyzed nsp12 subunits ranges from 51 to 96%, the arginine bracket is 100% conserved across all investigated coronaviruses. This conservation is also reflected at the structural level (*SI Appendix*, Fig. S10), suggesting that HeE1-2Tyr may also be able to bind to RdRp of various coronaviruses and inhibit their activity.

## Discussion

HeE1-2Tyr was initially discovered as a flavivirus inhibitor (31) that also inhibits SARS-CoV-2 RdRp (34). However, there was no understanding of the molecular mechanism of how HeE1-2Tyr binds and inhibits SARS-CoV-2 RdRp, which prevented further development of HeE1-2Tyr derivatives by structure-guided methods. In this study, we show that HeE1-2Tyr is a competitive RdRp inhibitor that prevents polymerase activity by displacing the RNA from RdRp. The atomic structure of HeE1-2Tyr bound to SARS-CoV-2 RdRp shows that HeE1-2Tyr binds as a stack of three molecules to the RNA binding site of RdRp. The compound stack is stabilized by a bracket of three arginine residues in nsp12 that are crucial for RNA binding and highly conserved across coronaviruses.

The functional importance of the arginine bracket for RNA binding suggests that mutations in SARS-CoV-2 RdRp that render the enzyme insensitive to HeE1-2Tyr will lead to a loss of viral fitness. This creates a high resistance barrier making it unlikely that HeE1-2Tyr-resistant RdRp variants evolve easily. Due to the high importance of the arginine bracket for RdRp function, the HeE1-2Tyr binding site is also structurally conserved. We therefore propose that HeE1-2Tyr or improved derivatives could bind to the same binding site in RdRp of other coronaviruses, such as the human pathogenic MERS-CoV, SARS-CoV-1, and the seasonal coronaviruses CoV-OC43 or CoV-NL63. Thus, HeE1-2Tyr is a promising starting molecule for the development of a potential pan-coronavirus drug.

Stacking of three small molecule inhibitors inside a binding site is rather unusual but has been reported before (48, 49). In one study, a triple stack of a polyphenolic ligand bound to an acetylcholine-binding protein was described (48). The stack of ligand molecules is stabilized by intercompound π–π stacking interactions, which is similar to what we observe for the stack of HeE1-2Tyr. Additionally, the structure of a helical tau filament bound to stacked PET ligands was reported, in which the stack is also stabilized by internal π–π interactions (49). However, binding of a stack of multiple compound molecules to a target protein is a rare binding mode that needs to be studied further. In the future, such binding modes could be exploited in order to increase the surface area of small molecule inhibitors. This would potentially allow rather small compounds to interfere with large interfaces, such as protein–protein and protein-nucleic acid interactions.

Binding of several ligands to a target protein can cause cooperativity, usually because one compound influences the affinity for another, for example by modulating the protein conformation (50). We also observed strong positive cooperativity in our biochemical characterization of HeE1-2Tyr-mediated RdRp inhibition. Considering that HeE1-2Tyr binds to RdRp as a stack of three molecules that interact with each other, the cooperativity can be readily explained. We propose that once HeE1-2Tyr_1_ binds to RdRp, a new binding interface for additional HeE1-2Tyr molecules is created. The intercompound interactions will then further stabilize incoming HeE1-2Tyr molecules, explaining how binding of one molecule might increase the affinity for the others. This mode of positive cooperativity is partly built into the small molecule compound and therefore does not require a large conformational change of the protein.

In contrast to the stack of three HeE1-2Tyr molecules bound to SARS-CoV-2 RdRp, the compound binds as a monomer to the Dengue virus RdRp (31). The RdRp of Dengue and SARS-CoV-2 are very distinct with a sequence identity of only 20%, thus their active sites and RNA binding are expected to differ. Indeed, the only common feature of the HeE1-2Tyr binding modes is that the molecule binds in the RNA binding site of both RdRps. However, the authors acknowledge that the crystal structure of the Dengue virus RdRp in complex with HeE1-2Tyr does not explain their biochemical data. This raises the possibility that stacking or an alternative binding site of HeE1-2Tyr inside the Dengue virus RdRp may have been missed due to the use of protein crystallography as a structure determination method (31).

Until now, the structure–activity relationship (SAR) of HeE1-2Tyr and RdRp inhibition was unknown, preventing the rational design of more potent derivatives. Initial attempts to modify HeE1-2Tyr affected the solubility of the compound, but did not provide clear insights into the SAR (34). A recent study attempted to simplify HeE1-2Tyr by replacing the pyridobenzothiazole core with pyridone or thiazolopyridone (51). Interestingly, both truncations of the heterocyclic core resulted in significantly less potent derivatives, which can be readily explained by the loss of intercompound π–π stacking interactions as observed in our structure. This underlines the importance of understanding the mode of action and therefore, future attempts to improve HeE1-2Tyr will benefit from our atomic structure.

Altogether, we present biochemical data and the cryo-EM structure of SARS-CoV-2 RdRp bound to a stack of three HeE1-2Tyr molecules, revealing that HeE1-2Tyr inhibits SARS-CoV-2 RdRp by competing with RNA binding. The identification of the unusual binding mode of HeE1-2Tyr and the high conservation of the binding site across coronaviruses make this molecule a potent candidate for future structure-based pan-coronavirus drug design.

## Methods

### Protein Preparation.

Preparation of SARS-CoV-2 RdRp, composed of nsp12, nsp7, and two copies of the nsp8 subunits, was carried out as described by Madru et. al. (37), with small adaptations. In brief, the RdRp expression plasmid (Addgene 165451) was used to transform *Escherichia coli* BL21(DE3) RIL. Cells were grown at 30 °C and 150 rpm shaking in LB media supplemented with Kanamycin until an optical density at 600 nm of 0.4 was reached. Then the temperature was reduced to 18 °C, and cells were cooled down for one hour. Protein expression was induced with 0.05 mM IPTG, and cells were further incubated for 16 h at 18 °C. After harvesting, the cells were resuspended in lysis buffer [50 mM Na-HEPES pH 8.0, 500 mM NaCl, 5% (vol/vol) glycerol, 30 mM imidazole pH 8.0, 5 mM β-mercaptoethanol, 0.284 μg ml^−1^ leupeptin, 1.37 μg ml^−1^ pepstatin, 0.17 mg ml^−1^ PMSF and 0.33 mg ml^−1^ benzamidine]. Cells were lysed using a sonicator (80% intensity, 10 s on, 50 s off, total time 2 min). The lysate was clarified by centrifugation at 74,766 g for 45 min, followed by filtration through a 0.45-μm filter (Amicon Ultra centrifugal filter, Merck). The protein was bound to HisTrap HP prepacked columns (GE Healthcare) preequilibrated in lysis buffer, washed with 10 column volumes (CV) lysis buffer and eluted with a gradient of 0 to 100% lysis to elution buffers [50 mM Na-HEPES pH 8.0, 500 mM NaCl, 5% (vol/vol) glycerol, 500 mM imidazole and 5 mM β-mercaptoethanol] over 30 CV. Fractions containing RdRp were pooled and fivefold diluted with dilution buffer (50 mM Na-HEPES pH 8.0, 5% (vol/vol) glycerol, and 5 mM β-mercaptoethanol) to reach a final NaCl concentration of 100 mM. The protein solution was applied to a HisTrap Q prepacked column preequilibrated in application buffer (50 mM Na-HEPES pH 8.0, 150 mM NaCl, 5% (vol/vol) glycerol, and 5 mM β-mercaptoethanol) to remove impurities, free nsp8 and nsp7/8. Bound RdRp was washed with 10 CV application buffer and then eluted with a gradient of 0-100% application to elution buffers Q [50 mM Na-HEPES pH 8.0, 1 M NaCl, 5% (vol/vol) glycerol, and 5 mM β-mercaptoethanol] over 20 CV. Elution peaks were analyzed using a 4 to 12% Bis-Tris SDS-PAGE. Peaks containing RdRp without any significant contamination were pooled and concentrated to 60 μM using a 10 kDa molecular weight cutoff Amicon Ultra Centrifugal Filter (Merck). During the concentration procedure, a buffer exchange to storage buffer [20 mM Na-HEPES pH 8.0, 300 mM NaCl, 5% (vol/vol) glycerol, 1 mM MgCl_2_ and 1 mM Tris(2-carboxyethyl)phosphine (TCEP)] was performed by diluting RdRp in storage buffer 5 times. The concentrated and buffer-exchanged protein was then aliquoted, flash-frozen in liquid nitrogen, and stored at −70 °C until further usage.

### RNA Extension Assays.

Preparation of SARS-CoV-2 coexpressed RdRp was carried out as described above, and preparation of individually expressed RdRp subunits was carried out as previously described (5). The RNA scaffold was designed according to published SARS-CoV-2 RNA extension assays (5, 12). All assays were performed with a single-stranded looped RNA scaffold containing a 6-carboxyfluorescein (FAM) fluorescent label at the 5’ end (Fig. 1*B*) purchased from Integrated DNA Technologies (IDT). The RNA sequence used for the assays was 5´-6-FAM/rUrUrUrUrCrA rUrGrCrArCrCrGrCrGrUrArGrUrUrUrUrCrUrArCrGrCrG-3´. The RNA template-product hairpin duplex allowed for RNA extension by 11 nucleotides.

For the HeE1-2Tyr dose curve, the final concentrations of RdRp and RNA in the reaction were 0.125 µM. The final HeE1-2Tyr concentrations ranged from 0.625 µM to 20 µM. As HeE1-2Tyr stocks were dissolved in DMSO, the DMSO concentration in the assays was held constant at 0.2%.

RNA was annealed in annealing buffer (50 mM NaCl, 10 mM Na-HEPES pH 7.5) by heating it to 75 °C for 1 min and gradually cooling to 4 °C. Annealed RNA was diluted in 50 mM NaCl reaction buffer (50 mM NaCl, 25 mM Na-HEPES pH 7.5, 2 mM MgCl_2_, 5 mM β-mercaptoethanol), and RdRp was diluted in 300 mM NaCl reaction buffer (300 mM NaCl, 25 mM Na-HEPES pH 7.5, 2 mM MgCl_2_, 5 mM β-mercaptoethanol). The annealed RNA and RdRp were combined, and the salt concentration of the reaction was adjusted to 50 mM NaCl using reaction buffer without NaCl (25 mM Na-HEPES pH 7.5, 2 mM MgCl_2_, 5 mM β-mercaptoethanol). HeE1-2Tyr and DMSO controls were diluted in 300 mM NaCl reaction buffer and then added to the annealed RNA and RdRp. The salt concentration of the reaction was adjusted to 50 mM NaCl using reaction buffer without NaCl. Annealed RNA, RdRp, and compound were then incubated for 30 min at 30 °C.

For comparing RdRp from different expression protocols (*SI Appendix*, Fig. S1) and mutants tests (*SI Appendix*, Fig. S9), RNA extension reactions contained RNA (0.4 µM), and either coexpressed RdRp (0.0125 µM to 3.2 µM) or individually expressed nsp12 (0.0125 µM to 3.2 µM) with threefold molar excess each of nsp8 (0.0375 µM to 9.6 µM) and nsp7 (0.0375 µM to 9.6 µM) in 50 mM NaCl reaction buffer. Reactions were incubated for 30 min at 30 °C to allow RdRp to bind RNA.

For all extension assay tests, RNA extension was started by the addition of NTPs (150 µM UTP, GTP, and CTP, and 300 µM ATP) in 50 mM NaCl reaction buffer. After 5 min of incubation at 30 °C, the reactions were stopped with 2 × stop buffer (6.4 M urea, 50 mM EDTA pH 8.0, 1 × TBE buffer). RNA products were resolved on 15% denaturing polyacrylamide-urea gels in 1 × TBE running buffer and visualized using a Typhoon 95000 FLA Imager (GE Healthcare Life Sciences). Bands were quantified with Image Lab (Bio-Rad). The fraction of extended RNA was calculated by obtaining the ratio of the band intensity (with background subtracted) of the RNA product strand to the combined adjusted intensity of the RNA substrate and product strands. For the HeE1-2Tyr dose curve, the fraction of RNA extended in the HeE1-2Tyr samples was normalized to that of the DMSO control. The obtained data were plotted and analyzed with GraphPad Prism version 9.

### RNA Fluorescence Polarization Assay.

The RNA fluorescence polarization (FP) assay was performed with the same single-stranded looped RNA scaffold as was used for the RNA extension assay. To study potential interactions of the RNA with its binding partners, the FP signal of the 6-carboxyfluorescein (FAM) label at the 5’ RNA end was utilized. Measurements of fluorescence and FP were performed with an Infinit M1000Pro plate reader (Tecan Group Ltd., Switzerland), with excitation and emission wavelengths of 470 nm (±5 nm) and 517 nm (±5 nm), respectively. All reactions were performed in assay buffer (20 mM Na-HEPES pH 8.0, 300 mM NaCl, 1 mM MgCl_2_, 5 mM β-mercaptoethanol) with a final reaction volume of 20 µl per well. Black 384-well nonbinding microplates (Greiner Bio-One, Germany) were used to record data.

For the assay development 25 nM of RNA was incubated with increasing concentrations of the respective protein (*SI Appendix*, Fig. S2). The reactions were incubated for 15 min at room temperature (RT); subsequently, the FP was measured. To remove background FP signal caused by the RNA itself, the delta of the FP signal was calculated by subtracting the mean FP of RNA only samples from each sample containing RNA and protein. Samples were measured in independent triplicates.

For the FP assay HeE1-2Tyr dose curve, 25 nM of RNA was incubated with 125 nM RdRp and increasing concentrations of HeE1-2Tyr (Fig. 2*A*). The final HeE1-2Tyr concentrations ranged from 0.625 µM to 20 µM, with 0.2% DMSO in all reactions. The reactions were incubated at RT for 30 min; subsequently, FP was measured. The delta FP was calculated as stated above and then normalized to the DMSO sample, resulting in relative FP signal. Samples were measured in independent triplicates.

For the FP assay shown in Fig. 2*B*, the order of RNA and HeE1-2Tyr was varied by preforming RdRp or preincubating RdRp and HeE1-Tyr. For preformed samples, RdRp was allowed to form a complex with RNA for 5 min at RT before HeE1-2Tyr was added. For preincubated samples, RdRp and HeE1-2Tyr were incubated together for various timepoints before RNA was added. Time of compound exposure is indicated in the figure legend. RdRp and RNA interact within seconds, making exact quantification impossible due to technical reasons), and the time of exposure to RNA had no significant influence. The delta FP was calculated as stated above and then normalized to the according DMSO control (DMSO controls performed for each condition and timepoint separately). Samples were measured in independent triplicates.

For the experiment shown in *SI Appendix*, Fig. S3, RdRp was performed with RNA for 5 min at RT, and then different HeE1-2Tyr concentrations were added. The FP signal was measured every 60 s for a total of 60 min. The delta FP was calculated as stated above and then normalized to the DMSO control of that specific timepoint. Samples were measured in independent triplicates, and the mean ± SD is reported in the figure.

The FP assay for mutant RdRps, shown in Fig. 5, utilizes the same protocol as the assay development experiment (*SI Appendix*, Fig. S2), with a deviation in the RdRp proteins used (3 K and 3A mutant instead of wilt type RdRp).

### Mass Photometry.

Mass photometry measurements were performed on a Refeyn TwoMP mass photometer using Refeyn AcquireMP and DiscoverMP software (both v. 2.2) as described (52) with minor modifications. For each measurement, 15 µL of buffer was added to the gasket, and the autofocus function was switched on. After successful focusing, 5 µL of prediluted protein sample was added, and the measurement was started. The resulting movies of 60Â s were then analyzed and graphed in the DiscoverMP software. For each measurement, at least 5,000 events were recorded and analyzed. Each condition was measured in independent triplicates.

For the comparison of RdRp preparation methods shown in *SI Appendix*, Fig. S4, RdRp was either coexpressed as stated above or reconstituted from individual subunits as previously described (5). 3 µM coexpressed RdRp or 1 µM nsp12, 3 µM Nsp7, and 3 µM nsp8 were incubated in SEC buffer [20 mM Na-HEPES pH 8.0, 300 mM NaCl, 5% (vol/vol) glycerol, 1 mM MgCl_2_, 1 mM TCEP] for 30 min at 30 °C. The samples were then cooled down to 4 °C and kept on ice. Shortly before the measurements, proteins were diluted with SEC buffer to 40 nM, and each sample was applied to the mass photometer as stated above.

In Fig. 5 *B* and *D*, the stability of RdRp was investigated in the presence of HeE1-2Tyr or RNA. Therefore, 300 nM coexpressed wild-type RdRp or 3 K mutant were incubated in assay buffer (50 mM NaCl, 25 mM Na-HEPES pH 7.5, 2 mM MgCl_2_, 5 mM β-mercaptoethanol) for 60 min at 4 °C with various concentrations of HeE1-2Tyr or with 1.25 µM RNA template-product hairpin (same sequence as was used in the RNA extension assay and FP assay but without FAM). HeE1-2Tyr concentrations ranged from 0.625 to 20 µM, and DMSO was kept constant at 0.2%.

After the incubation, mixtures were diluted to 40 nM, and each sample was applied to the mass photometer as stated above. To analyze the abundance of RdRp, counts of events corresponding to the molecular weight of RdRp were divided by the total number of events. The abundance of RdRp in each sample was then normalized to the DMSO control, resulting in the relative abundance of RdRp.

### Cryo-EM Sample Preparation and Data Collection.

Cryo-EM sample preparation and data collection were performed as described (12) with minor adaptations. Briefly, 6.6 µM coexpressed RdRp was incubated with 50 µM HeE1-2Tyr or 0.5% DMSO for 30 min at 4 °C. 3 µl of the reaction were mixed with 0.5 µl of octyl β-D-glucopyranoside (0.003% final concentration) and applied to freshly glow-discharged UltrAufoil® R 1.2/1.3, 300 mesh grids (Quantifoil). The grids were blotted for 6 s with blot force 5 using a Vitrobot Mark IV instrument (Thermo Fischer Scientific) at 4 °C and 95% humidity and plunge-frozen in liquid ethane.

Cryo-EM data were collected with SerialEM (53) on a Titan Krios transmission electron microscope (Thermo Fisher Scientific) operated at 300 keV. Inelastically scattered electrons were filtered out with a GIF Quantum energy filter (Gatan) using a slit width of 20 eV. Images were acquired using a K3 direct electron detector in counting mode (nonsuper resolution) at a nominal magnification of ×105,000, resulting in a calibrated pixel size of 0.834 Å px^−1^. Images were exposed for a total of 2.43 s with a dose rate of 23.84 e^−^ px^−1^ s^−1^, resulting in a total dose of 57.92 e^−^/Å2 that was fractionated into 80 frames. Motion correction, CTF estimation, particle picking, and extraction were performed using Warp (54). A total of 11,115 and 11,230 movies were collected for RdRp-HeE1-2Tyr and RdRp-DMSO samples, respectively.

### Cryo-EM Data Processing and Structural Modeling.

For the RdRp-HeE1-2Tyr dataset, 4.17 million particles were extracted using Warp (54) 1.0.9. Particles were imported to cryoSPARC (38) and subjected to two-dimensional (2D) classification. 2D classes representing contamination or broken particles were selected and used for calculating three ab initio structures. All particles were then used for supervised 3D classification against four references, where three originated from the ab initio reconstruction of contamination and broken particles and one was a previous RdRp complex structure [EMD-30572 (27)]. The class containing RdRp yielded ~1.6 million particles, which were subjected to homogeneous 3D refinement. The refined particles were then exported to RELION 3.1. To separate fully assembled RdRp from smaller subcomplexes, particles were 3D-classified without image alignment (T = 4, six classes) using a mask around RdRp that omitted the nsp8 sliding poles. This resulted in two promising classes that appeared to contain fully assembled RdRp particles. However, the viewing angles of class 2 were significantly better (red arrow in *SI Appendix*, Fig. S5), hence we continued with ~300,000 particles from class 2. To clearly separate particles with and without the extra compound density, we performed focused 3D classification without image alignment but with a mask around the active site of RdRp (mask: low pass filtered extra density with 20 pixel extension). This resulted in ~200,000 particles with extra density that we focus-refined to an overall resolution of 3.0 Å. Local resolution was estimated with Relion 3.1 using a kernel size of 10 Å. For the RdRp-DMSO dataset, 3.06 million particles were extracted. Further processing was similar to the first dataset with the exception that the focused classification of the active site was not performed. This resulted in ~200,000 particles of RdRp-DMSO that we refined to an overall resolution of 3.1 Å. The angular distribution and 3DFSC of both maps were calculated according to Tan et al. (55)

In order to calculate a difference map, the maps of both datasets were low-pass filtered to 3.2 Å using Relion image handler and then intensity scaled using ChimeraX (56). The RdRp-DMSO map was then subtracted from the map derived from the RdRp-HeE1-2Tyr sample, and dust removal (threshold 14) was applied to the resulting difference map to remove noise.

Atomic models were built using the previously published SARS-CoV-2 RdRp structure [PDB 6M71 (40)] as a starting model. The model was first rigid-body-fitted into the density and then adjusted with ISOLDE (57) flexible fitting. Based on the high local resolution of the extra density and the three observed layers, three HeE1-2Tyr molecules were manually placed into the extra density. HeE1-2Tyr molecules were then adjusted with the interactive molecular dynamics package from ISOLDE, which allowed us to unambiguously model three stacked molecules of HeE1-2Tyr. The protein chains were then subjected to phenix.real_space_refine. Restraints for HeE1-2Tyr molecules were generated in phenix.elbow (58), and the model quality was assessed using MolProbity within Phenix (59), which revealed excellent stereochemistry (*SI Appendix*, Table S1). All figures were prepared with ChimeraX (56).

### Ligand Interactions.

To analyze potential ligand–protein and ligand–ligand interactions, the Arpeggio tool (60) and LigPlot + v.2.2 (61) were used. Proposed interactions were then interpreted based on distances measured with ChimeraX (56).

### Sequence Conservation.

To analyze the sequence conservation of residues of the arginine bracket, a multiple sequence alignment (MSA) of ORF1ab across corona viruses was performed. Therefore, we assembled a small database of corona virus representatives from all four coronavirus families using the Virus Variation Resource (62). An MSA was then calculated using Clustal Omega (63, 64). Results were visualized with Jalview (65).

## Supplementary Material

Appendix 01 (PDF)

Dataset S01 (XLSX)

## Data Availability

The cryo-EM reconstructions and structure coordinates for the RdRp–HeE1-2Tyr structure have been deposited with the Electron Microscopy Database (EMDB) under accession code EMD-52704 (66) and with the Protein Data Bank (PDB ID 9I81) (67). Source data are provided with this paper.
