# Supplementary material for: Structural basis of SARS-CoV-2 polymerase inhibition by nonnucleoside inhibitor HeE1-2Tyr
Source: Proc Natl Acad Sci U S A. 2025 Mar 4;122(10):e2419854122. doi: 10.1073/pnas.2419854122 (PMC11912441; doi:10.1073/pnas.2419854122)
Supplement: Supplementary file 1 — Appendix 01 (PDF) [file pnas.2419854122.sapp.pdf]

PNAS

Supporting Information for

Structural basis of SARS-CoV-2 polymerase inhibition by non-nucleoside inhibitor HeE1-2Tyr

Florian Kabinger[1], Valerie Doze[1], Jana Schmitzová[1], Michael Lidschreiber[1,*], Christian Dienemann[1,*], Patrick Cramer[1,*]

Michael Lidschreiber, Christian Dienemann, Patrick Cramer
Email: michael.lidschreiber@mpinat.mpg.de, christian.dienemann@mpinat.mpg.de, patrick.cramer@mpinat.mpg.de

This PDF file includes:

Figures S1 to S10
Table S1

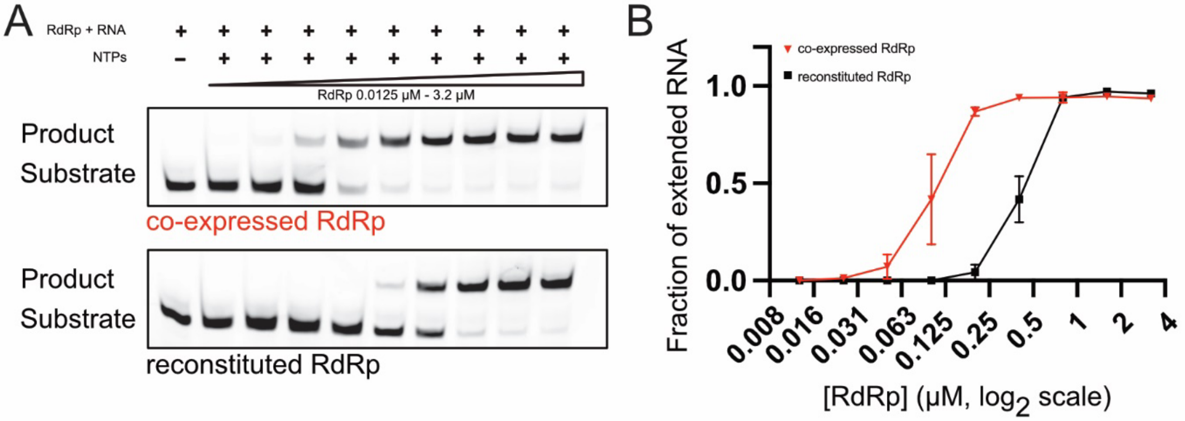

Fig. S1: Comparison of the enzymatic activity of RdRp co-expressed and reconstituted from individual subunits

A, Gel-based comparison of the enzymatic activity of RdRp co-expressed (top, red) and reconstituted from individual subunits (bottom, black). Minimal RNA substrate was incubated with NTPs and increasing concentration of the according RdRp (0.0125μM – 3.2μM, two-fold serial dilution); reaction products were separated on a denaturing acrylamide gel. **B**, Quantification of the experiment in (**A**) after triplicate measurements. Mean ± s.d. of the fraction of extended RNA are plotted; RdRp concentration is represented on a logarithmic scale with base 2.

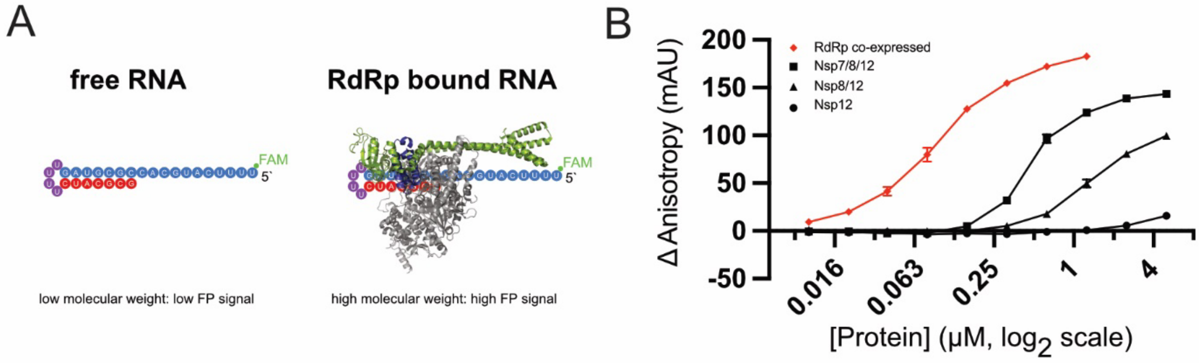

Fig. S2: Development of a fluorescence polarization (FP) assay to monitor the interaction between RdRp and RNA

A, Principle of the FP assay. RNA harbors a 5′ 6-carboxyfluorescein (FAM) label (green). Free RNA has a low molecular weight, low hydrodynamic radius and consequently a low FP signal whereas RNA bound to RdRp behaves the opposite. **B**, Quantification of the fluorescence anisotropy of RNA exposed to increasing concentrations of co-expressed RdRp (red diamonds), RdRp reconstituted from individual subunits (black squares), nsp8 and nsp12 (black triangles) and nsp12 alone (black circles). Mean ± s.d. of independent triplicate measurements are shown, protein concentration is represented on a logarithmic scale with base 2.

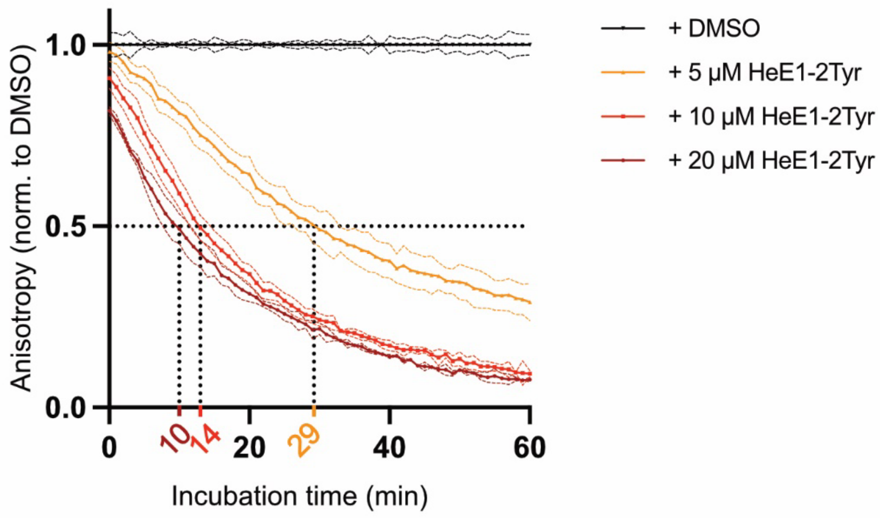

Fig. S3: Effect of HeE1-2Tyr on the RdRp-RNA interaction over time
Real-time quantification of the fluorescence anisotropy of preformed RdRp (125 nM) exposed to 5 µM (orange), 10 µM (red), and 20µM (dark red) HeE1-2Tyr relative to the DMSO control (black). Mean ± s.d. of independent triplicate measurements are shown; measurement interval: 1 minute. 50% reduction of the anisotropy relative to the DMSO control indicated by dashed lines and color-coded values on x-axis.

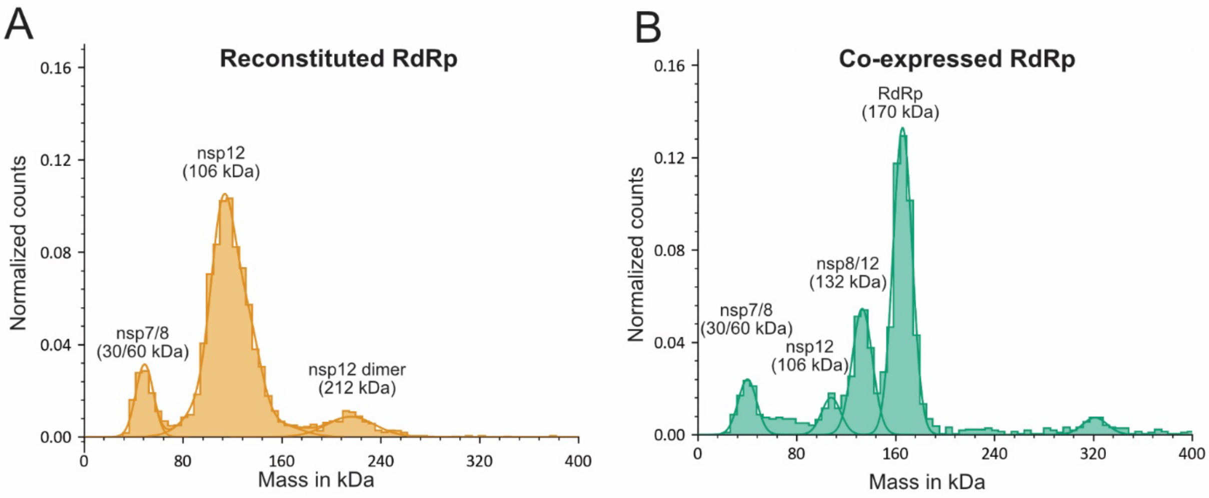

Fig. S4: Mass photometry-based analysis of RdRp reconstituted from individual subunits and co-expressed

A, RdRp reconstituted from individual subunits (yellow). **B**, Co-expressed RdRp (blue). Relevant peaks are annotated and labeled with the name of the respective proteins and the expected molecular weight of their complexes. The most abundant form of nsp7/8 is a hetero dimer or tetramer, giving rise to a MW distribution representing a mixture of both oligomerization states.

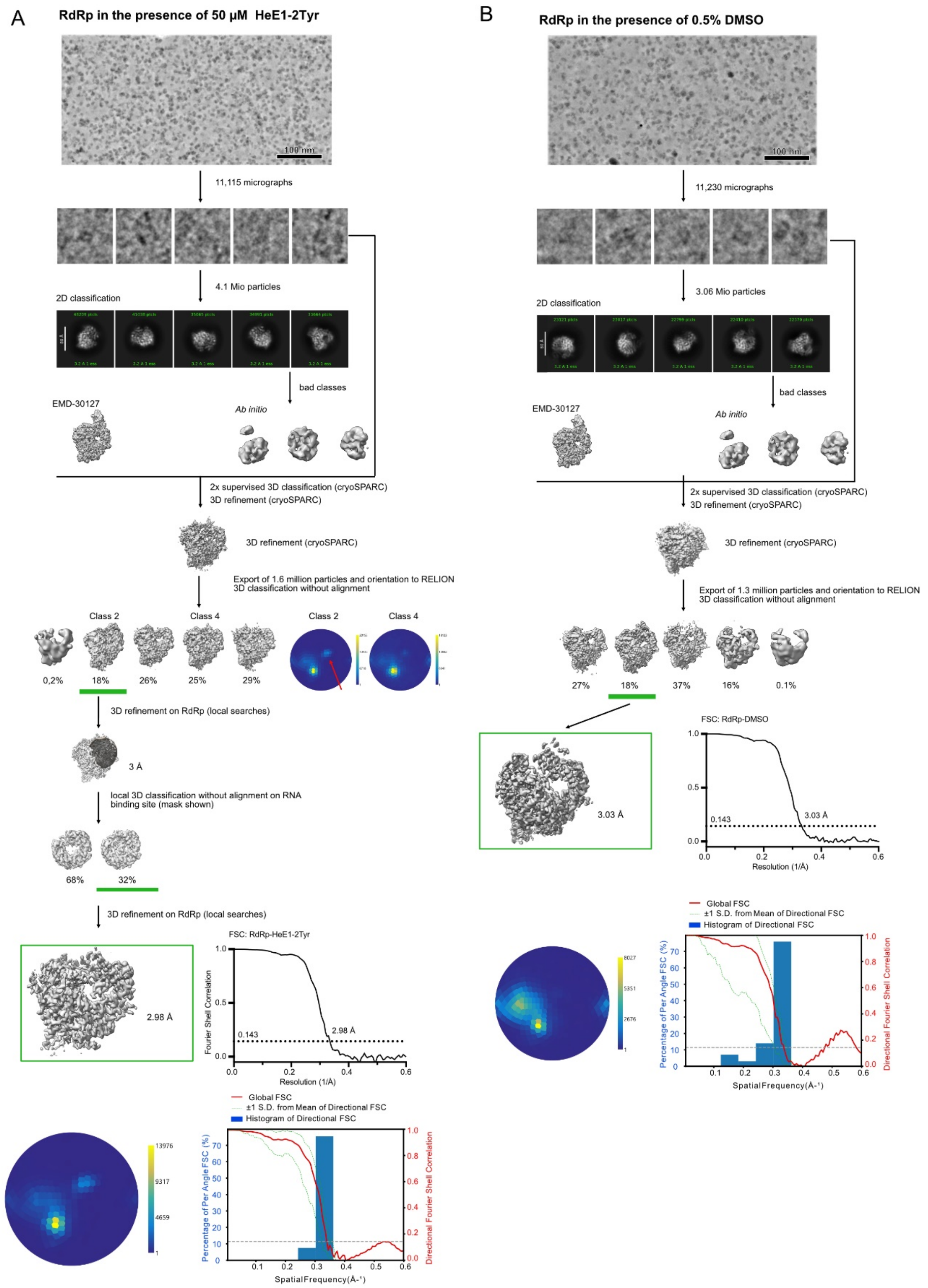

Fig. S5: Cryo-EM data processing trees and quality of reconstructions

A, Cryo-EM data processing tree for RdRp in the presence of 50 µM HeE1-2Tyr. Scale bar, 100 nm. FSC plot, angular distribution and directional FSC calculated according to ref.[1]. Global resolution of RdRp-HeE1-2Tyr structure is 2.98 Å and sphericity is 0.903. (Legend continued on next page)

B, Cryo-EM data processing tree for RdRp in the presence of 0.5% DMSO. Scale bar, 100 nm. FSC plot, angular distribution and directional FSC calculated according to ref.[1]. Global resolution of RdRp-DMSO structure is 3.03 Å and sphericity is 0.854.

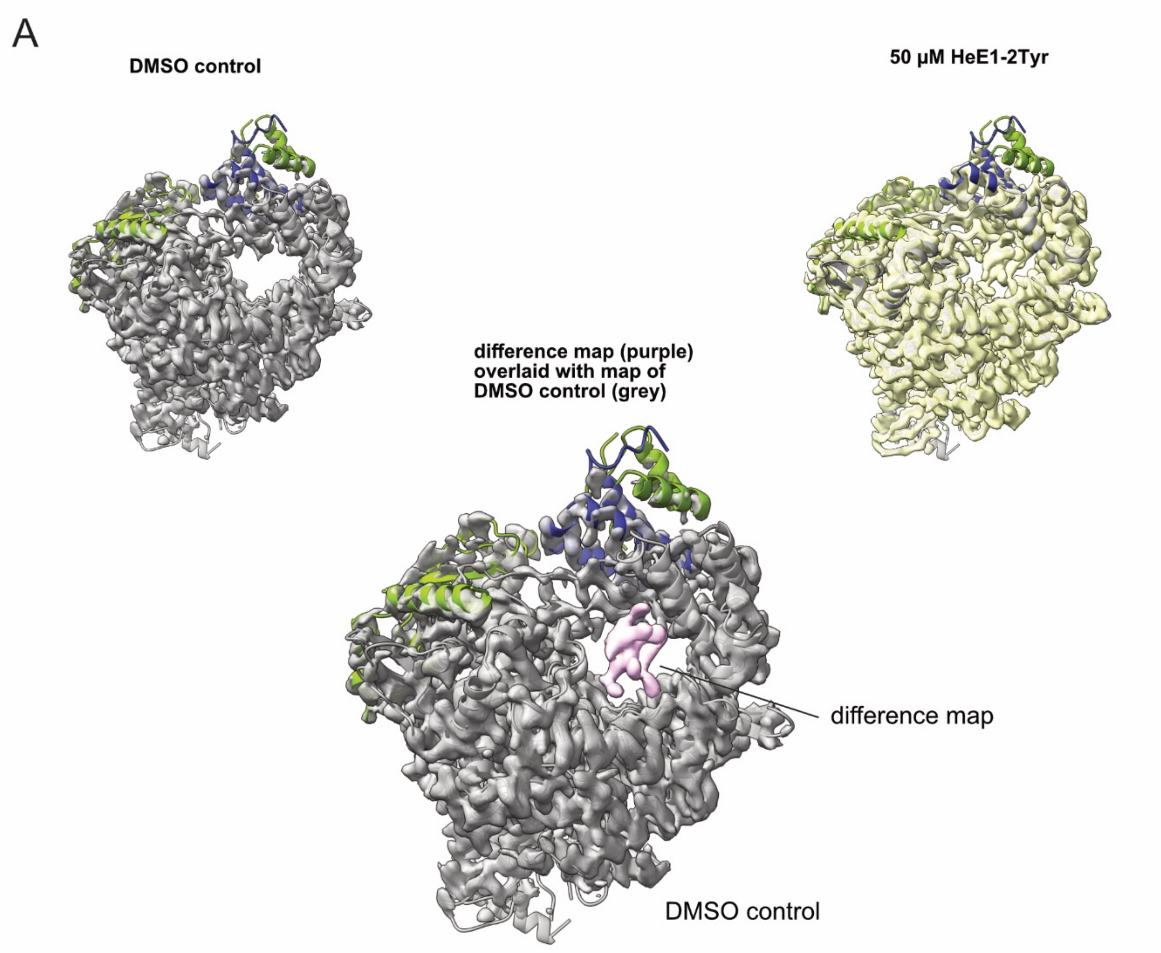

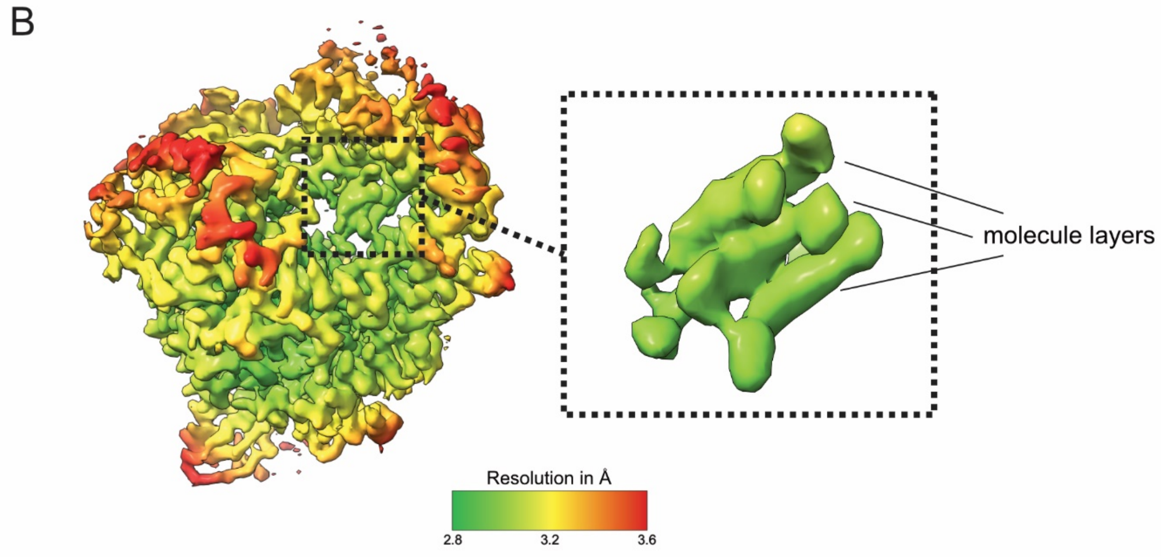

Fig. S6: Difference map and map quality

A, Reconstruction of the DMSO control (grey) and RdRp-HeE1-2Tyr (yellow) overlaid with atomic model of RdRp (PDB ID: 6M71[2]). Reconstructions are shown as semi-transparent grey density; RdRp subunits nsp7, nsp8 and nsp12 are in dark blue, green and gray. Difference map of RdRp-DMSO and RdRp-HeE1-2Tyr **r**econstructions (pink) overlaid with the reconstruction of the DMSO control; surface dust removal size threshold: 14. **B**, Local resolution of the RdRp-HeE1-2Tyr **r**econstructions with close-up on ligand density (dotted box).

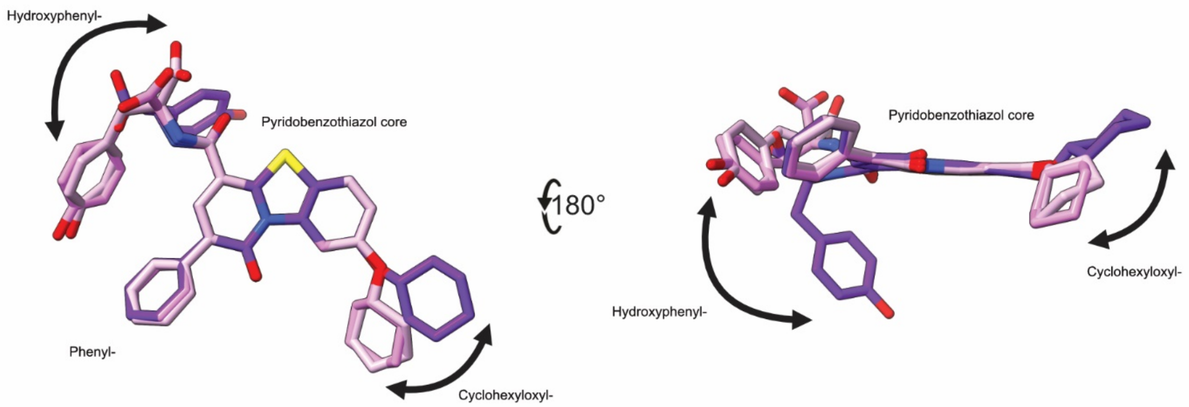

Fig. S7: Conformational changes of HeE1-2Tyr$_{1-3}$

HeE1-2Tyr molecules are superimposed and the conformational changes of the side chains are indicated by black arrows. HeE1-2Tyr$_{1-3}$ are shown in violet, pink and light red, respectively; relevant chemical groups labeled.

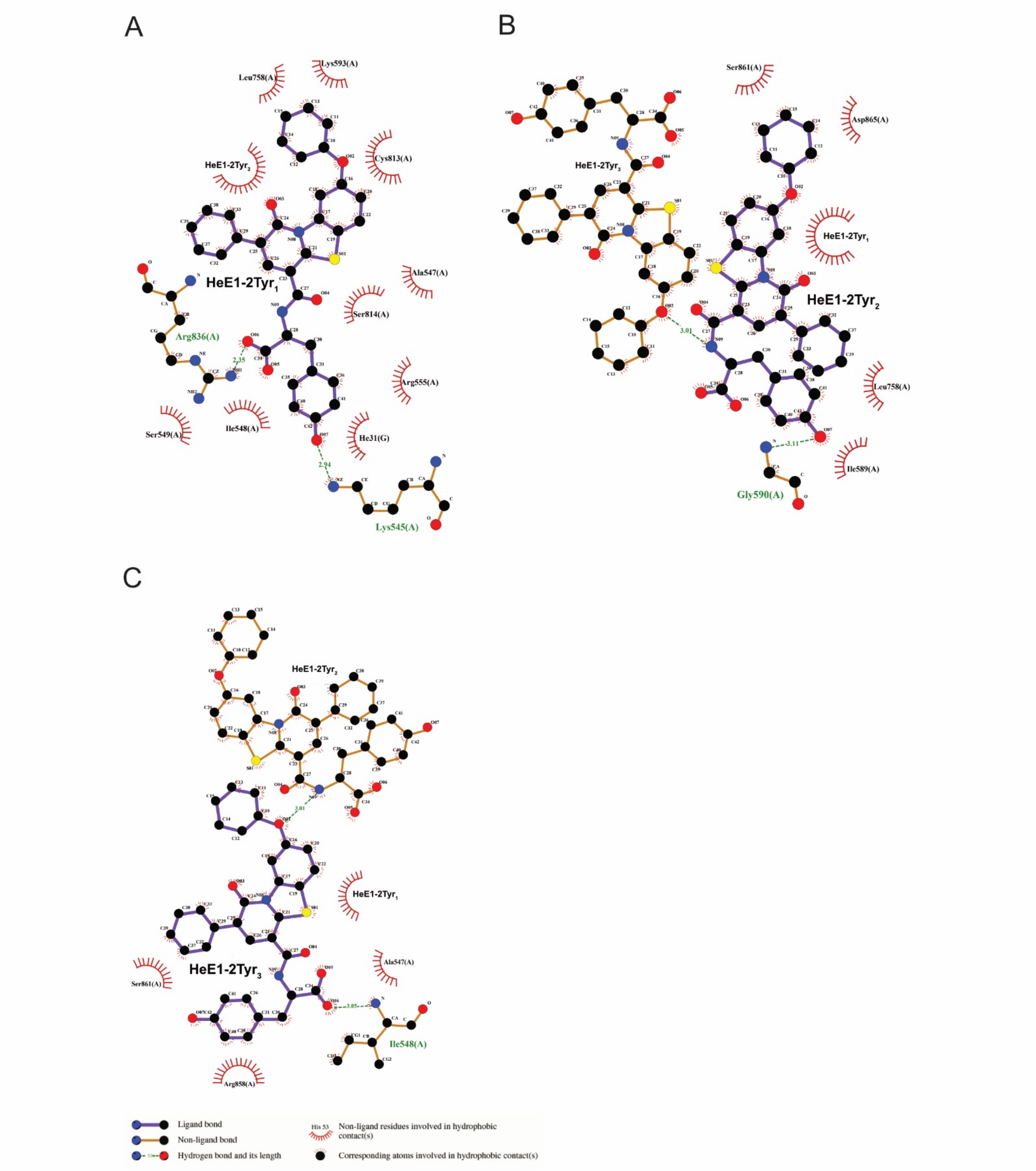

Fig. S8: Ligand interaction plot for HeE1-2Tyr$_{1\text{-}3}$

Ligand interaction plots for **A**, HeE1-2Tyr$_1$; **B**, for HeE1-2Tyr$_2$; **C**, for HeE1-2Tyr$_3$. Ligand interaction plots created with LigPlot + v.2.2[3]

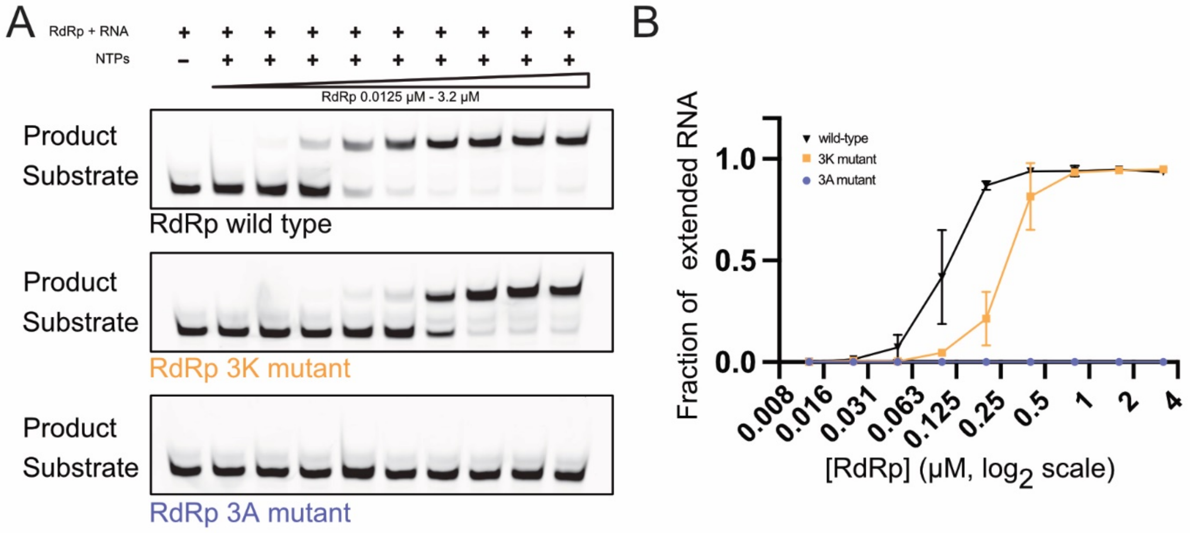

Fig. S9: Comparison of the activity of RdRp mutants

A, Gel-based comparison of the transcription activity of RdRp wild type (top), 3K mutant (middle, yellow) and 3A mutant (bottom, blue). Minimal RNA substrate was incubated with NTPs and increasing concentration of the according RdRps (0.0125µM – 3.2µM, two-fold serial dilution); reaction products were separated on a denaturing acrylamide gel. **B**, Quantification of the experiment in (**A**) after triplicate measurements. Mean ± s.d. of the fraction of extended RNA are plotted; RdRp concentration is represented on a logarithmic scale with base 2.

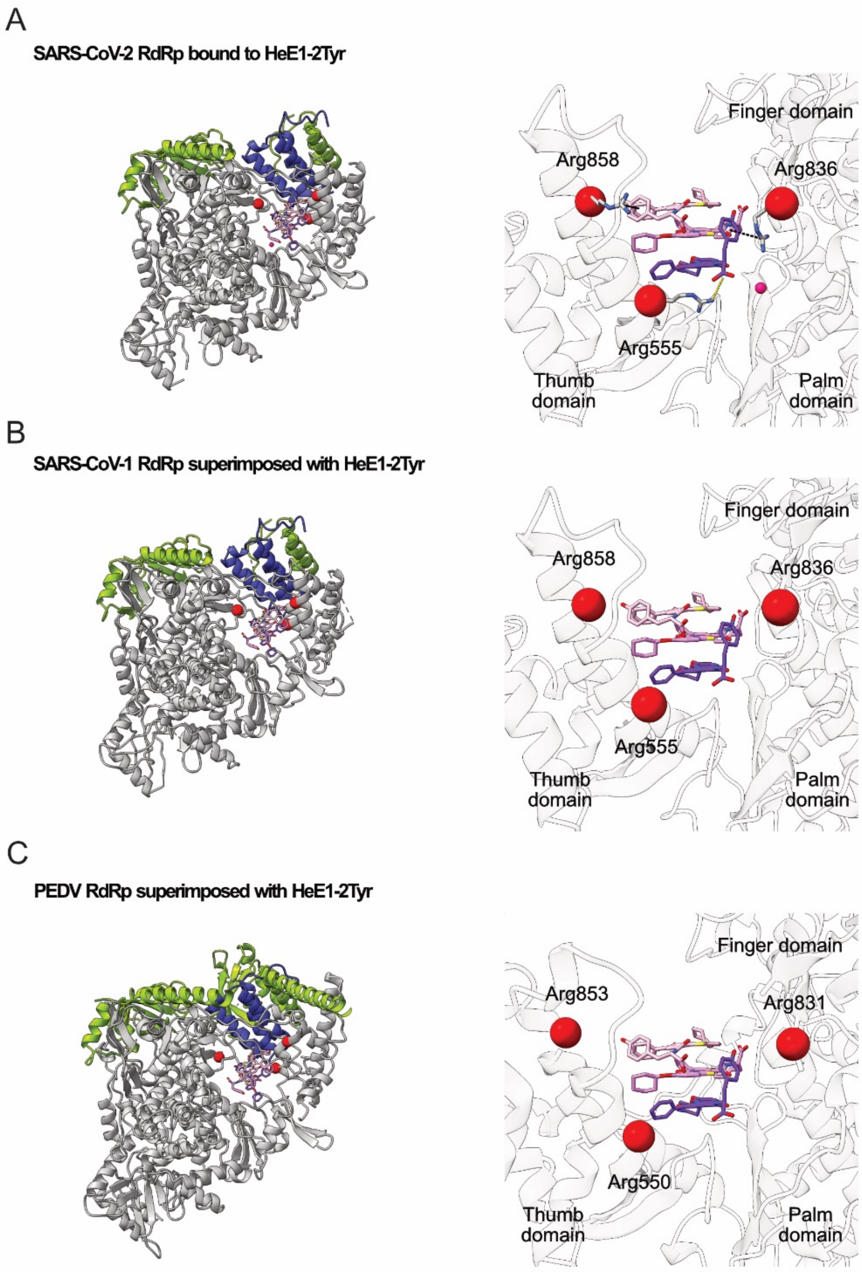

Fig. S10: Structural comparison of the RNA binding site in corona viruses

A, Cryo-EM structure of SARS-CoV-2 RdRp bound to HeE1-2Tyr. Active site ion indicated by magenta sphere; HeE1-2Tyr$_{1-3}$ are shown in violet, pink and light red, respectively; C-alpha atoms of Arg555, Arg837 and Arg858 depicted as red spheres; residues and RdRp domains are labeled. Close-up: HeE1-2Tyr binding site. **B**, Cryo-EM structure of SARS-CoV-1 RdRp (PDB ID: 6NUR[4]) superimposed with stack of three HeE1-2Tyr molecules from RdRp-HeE1-2Tyr structure. Same color code as **a;** c-alpha atoms of Arg555, Arg837 and Arg858 depicted as red spheres. **C**, Cryo-EM structure of PEDV RdRp (PDB ID:8URB[5]) superimposed with stack of three HeE1-2Tyr molecules from RdRp-HeE1-2Tyr structure. Same color code as (**A**) .C-alpha atoms of Arg550, Arg831 and Arg853 depicted as red spheres.

Table S1. Cryo-EM data collection, refinement and validation statistics

	SARS-CoV-2 RdRp bound to a stack of three HeE1-2Tyr molecules<br>(EMD-52704)<br>(PDB ID 9I81)
**Data collection and processing**	
Magnification	105,000
Voltage (kV)	300
Electron exposure (e–/Å^2)	57.9
Defocus range (μm)	0.4-2.2
Pixel size (Å)	0.834
Symmetry imposed	C1
Initial particle images (no.)	4,171000
Final particle images (no.)	189,000
Map resolution (Å)<br>FSC threshold	2.9<br>0.143
Map resolution range (Å)	2.8-5.2
**Refinement**	
Initial model used (PDB code)	6M71
Model resolution (Å)<br>FSC threshold	3.0<br>0.5
Map sharpening *B* factor (Å^2)	-40
Model composition<br>Non-hydrogen atoms<br>Protein residues<br>Ligands	 8740<br>1085<br>3
B factors (Å^2)<br>Protein<br>Ligand	 72.85<br>66.75
R.m.s. deviations<br>Bond lengths (Å)<br>Bond angles (°)	 0.014<br>0.772
Validation<br>MolProbity score<br>Clashscore<br>Poor rotamers (%)	 1.72<br>8.58<br>0.0
Ramachandran plot<br>Favored (%)<br>Allowed (%)<br>Disallowed (%)	 96.16<br>3.84<br>0.00

SI References

1. Tan, Y.Z. et al. Addressing preferred specimen orientation in single-particle cryo-EM through tilting. *Nat Methods* **14**, 793-796 (2017).
2. Gao, Y. et al. Structure of the RNA-dependent RNA polymerase from COVID-19 virus. *Science* **368**, 779-782 (2020).
3. Wallace, A.C., Laskowski, R.A. & Thornton, J.M. LIGPLOT: a program to generate schematic diagrams of protein-ligand interactions. *Protein Eng* **8**, 127-34 (1995).
4. Kirchdoerfer, R.N. & Ward, A.B. Structure of the SARS-CoV nsp12 polymerase bound to nsp7 and nsp8 co-factors. *Nat Commun* **10**, 2342 (2019).
5. Anderson, T.K., Hoferle, P.J., Lee, K.W., Coon, J.J. & Kirchdoerfer, R.N. An alphacoronavirus polymerase structure reveals conserved co-factor functions. *bioRxiv* (2023).
